# Pellagra: An Unusual Cause for Altered Mental Status

**DOI:** 10.7759/cureus.71315

**Published:** 2024-10-12

**Authors:** Jerome Gnanaraj, Waseem Khaliq, Susrutha Kotwal

**Affiliations:** 1 Internal Medicine, Johns Hopkins University School of Medicine, Baltimore, USA; 2 Medicine, Johns Hopkins University School of Medicine, Baltimore, USA

**Keywords:** approach to chronic diarrhea, niacin deficiency, pellagra, rare cause of altered mental status, uncommon rash

## Abstract

Pellagra, a rare disorder caused by niacin deficiency, manifests as 4Ds: dermatitis, diarrhea, dementia, and, if left untreated, death. Though it was thought to be eradicated from the United States after fortification with niacin, there have been concerns about its reemergence in specific high-risk populations such as chronic alcoholism, malabsorption, and anorexia nervosa. Here we present a patient with altered mental status who subsequently developed diarrhea during the course of hospitalization. After extensive workup, the final diagnosis was determined to be pellagra. The patient was administered niacin, resulting in subsequent improvement, and was discharged in stable condition from the hospital. Our case report highlights the importance of considering pellagra in the differential diagnosis for certain high-risk patients presenting with altered mental status as it has not been completely eradicated yet.

## Introduction

Pellagra is an uncommon disease resulting from a cellular deficiency of niacin and usually presents with symptoms such as dermatitis, diarrhea, and dementia [1]. Niacin is a water-soluble vitamin that is converted to nicotinamide-adenine-dinucleotide (NAD) and its phosphate (NADP). NAD is necessary for glycolysis and catabolism of protein and alcohol, and NADP is involved in cholesterol and fatty acid synthesis. Deficiency of these compounds inhibits the ability to repair cell damage and can affect cells with high turnover involving the skin, gastrointestinal tract, and brain [2]. Due to its rarity in developed countries, there is often a delay in both diagnosis and initiation of treatment. The current incidence of pellagra in the US is unknown, as there are only sporadic cases [3]. Untreated pellagra can eventually lead to the death of the patient [2]. Diagnosis can be easily missed if it is not actively considered during the clinical evaluation [4]. While nutritional deficiency was historically the primary cause of pellagra, in the developed world, this has mostly been eradicated with food fortification, and currently, secondary factors like chronic alcoholism, malabsorption, anorexia nervosa, and drug-induced niacin deficiency are some of the common reasons for patients developing niacin deficiency [4].

## Case presentation

A 68-year-old man with a past medical history of deep venous thrombosis, basal, and squamous cell skin cancer, adenocarcinoma of the colon (status post sigmoid resection and chemotherapy), and hypothyroidism was brought into the emergency department due to altered mental status over three weeks. Given that the patient was unable to provide details regarding his presentation, much of the medical history was acquired from the patient's family members. According to the family, the patient had been experiencing forgetfulness, confusion, and nausea/vomiting with poor appetite for approximately three weeks leading up to the presentation. Notably, there were no reports of fever, chills, or abdominal pain. Before the current presentation, the patient was alert, oriented, and independently managing activities of daily living. 

In the emergency room, the patient was afebrile with a heart rate of 106/minute, respiratory rate of 20/minute, and blood pressure of 102/70 mmHg, alongside an oxygen saturation of 97 percent on room air. Upon examination, he was oriented to himself but demonstrated lethargy and confusion. The rest of the physical exam was unremarkable. A computed tomography (CT) scan of the head without contrast revealed no acute intracranial pathology. The patient and family did not report any significant alcohol use. During the hospitalization, the patient's mental status continued to deteriorate with worsening delirium. On the fourth day, he experienced a high-grade fever and diarrhea. Additional history from the family revealed that for the past three weeks before the patient’s presentation to the hospital, he was having intermittent episodes of diarrhea. Stool cultures, ova and parasites, and stool Clostridium difficile tests yielded negative results. Meningitis was suspected, and broad-spectrum antibiotics were initiated. Blood cultures obtained before antibiotic administration remained negative, and lumbar puncture did not support a diagnosis of meningitis. Additional investigations, including thyroid stimulating hormone (TSH), human immunodeficiency virus (HIV), rapid plasma reagin (RPR), urinalysis, chest X-ray, and CT scans of the chest, abdomen, and pelvis, also yielded negative results. After completing the antibiotic course, the patient's mental status did not improve. Due to persistent delirium, a magnetic resonance imaging (MRI) of the brain was performed, and neurology consultation was sought. The MRI showed no acute intracranial pathology. Neurology recommended intravenous thiamine repletion and work-up for other potential vitamin and nutritional deficiencies. Therefore, methylmalonic acid (MMA), vitamin B12, homocysteine, vitamin E, copper, and niacin levels were sent. An electroencephalogram (EEG) did not show any seizure-like activity.

**Figure 1:**
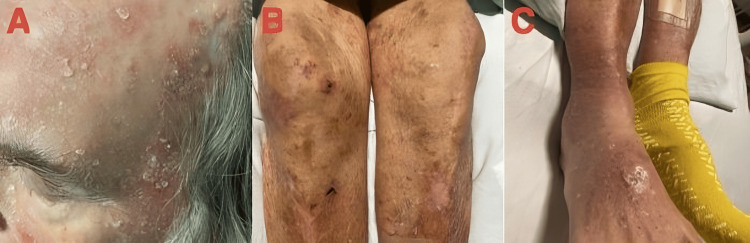
Diffuse erythematous scaly rash on the face (A) and lower extremities (B and C)

On day 10 of hospitalization, the patient exhibited a rash on his face, scalp, and extremities (Figure 1). This was thought to be due to seborrheic dermatitis. His oral mucosa and tongue examination were normal without any sign of stomatitis or glossitis. On day 12, the patient developed fever, worsening diarrhea, and shock, prompting a transfer to the ICU. In the ICU, vasopressors and hydrocortisone were initiated due to suspected adrenal insufficiency. Further laboratory results showed central adrenal insufficiency with low levels of adrenocorticotropic hormone (ACTH) and cortisol (Table 1). Endocrinology was consulted, and the patient was continued on repletion with hydrocortisone. Also, the levels of previously sent niacin and the metabolite nicotinamide were found to be low, further supporting the diagnosis of pellagra (Table 1). All other tests for vitamins and minerals were normal. He was started on niacin repletion at 250 mg every six hours, resulting in gradual symptomatic improvement. The patient's mental status continued to improve, as well as the rash and diarrhea. He was eventually discharged from the hospital in a stable condition. Subsequent follow-up visits with the primary care physician (PCP) revealed the patient to be oriented to place, person, and time, with a Mini-Mental State Examination (MMSE) score of 30/30. No diarrhea or rash was reported during follow-up appointments, indicating sustained improvement in his overall health.

**Table 1:** Important laboratory findings

Name of the test	Patient results	Reference range
Adrenocorticotropic hormone	Less than 5 pg/ml	6 to 50 pg/ml
Cortisol	0.8 mcg/dL	4.6 to 23.4 mcg/dL
Niacin	Less than 20 ng/ml	20 to 30,000 ng/ml
Nicotinamide	33 ng/ml	greater than 40 ng/ml

## Discussion

Niacin is present in various food sources, such as eggs, milk, beans, and fortified flour. It is absorbed in the small intestine and stored in the liver. Additionally, niacin can be synthesized in the human body from the essential amino acid tryptophan. As a water-soluble vitamin, niacin is crucial for oxidative phosphorylation and DNA regulation. The recommended daily allowance for niacin is 16 milligrams per day for adult males and 14 milligrams per day for females [5]. A deficiency of niacin can inhibit the repair of cell damage and affect tissues that have high cell turnover, like the skin, gastrointestinal (GI) tract, and the brain [2]. 

Pellagra, which translates to "rough skin" in Italian, gained recognition in the United States at the beginning of the 20th century. During the pellagra epidemic, approximately three million cases and about 100,000 deaths occurred over four decades [6]. However, the implementation of food fortification played a crucial role in nearly eradicating pellagra from the United States [7]. Clinical manifestations of pellagra include cutaneous, gastrointestinal, and neuropsychiatric symptoms. Cutaneous symptoms primarily appear in areas exposed to ultraviolet radiation, with the characteristic lesion known as Casal’s necklace involving the face and the neck. The dorsum of the hand and the extensor surface of the forearms can also be affected [8,9]. Gastrointestinal symptoms include nausea, vomiting, inflammation of the oral mucosa, glossitis, gastritis, and colitis. Intestinal inflammation induces diarrhea and malabsorption, potentially leading to malnutrition and cachexia, thereby exacerbating nutritional deficiencies. Niacin deficiency can cause a diverse array of neuropsychiatric symptoms, ranging from nonspecific symptoms like fatigue to peripheral neuropathy, weakness, depression, hallucinations, schizophrenia, delirium, and dementia [10,11]. It is noteworthy that the neurological symptoms observed in pellagra can mimic those of Wernicke's encephalopathy, as alcohol use is a common contributor to niacin deficiency [12,13]. Therefore, recognizing and addressing the varied clinical manifestations of pellagra is essential for accurate diagnosis and appropriate management. There has also been a case report of pellagra causing isolated neuropsychiatric symptoms without any GI or skin findings [12].

Despite reports suggesting the eradication of pellagra in developed countries, sporadic cases still occur and are often overlooked, leading to delayed diagnosis [12]. There is growing concern about the reemergence of pellagra among patients with anorexia and HIV infection [14,15]. Individuals affected by pellagra can experience delirium, which may progress to unconsciousness and ultimately death [15]. Therefore, early consideration of this diagnosis is crucial in the clinical course. While various biochemical tests, such as niacin, tryptophan, NAD, and NADP levels, could be informative, there is no single definitive test for pellagra [2,16]; therefore, pellagra is a clinical diagnosis. In our patient, the niacin result was available seven days after the sample was sent, emphasizing the importance of recognizing pellagra clinically and initiating empirical treatment rather than waiting for biochemical tests, as untreated pellagra can lead to fatal outcomes. The recommended treatment for pellagra is nicotinamide 300 milligrams daily in three to four divided doses [17]. Therapy should be continued until all symptoms show improvement. Encouraging the consumption of niacin-rich foods, including eggs, meat, fish, peanuts, and legumes, is also recommended as part of the overall management plan.

## Conclusions

Pellagra persists and may manifest in patients with specific risk factors, such as alcoholism, malabsorption, and anorexia nervosa. The potential for missed or delayed diagnosis underscores the importance of recognizing this condition, as its progression can lead to severe outcomes, including death. Our patient presented with altered mental status, delirium, rash, and diarrhea (albeit not at the same time) indicative of likely niacin deficiency. Treatment with niacin resulted in significant improvement across all these symptoms during the hospitalization, and the patient exhibited no evidence of these symptoms during follow-up visits with the PCP. This case report highlights the significance of considering niacin deficiency and other nutritional deficiencies in patients presenting with altered mental status. Early diagnosis and repletion can lead to the reversal of this condition, emphasizing the critical role of timely intervention. The diagnosis of pellagra relies on clinical features, and initiating treatment promptly is crucial in preventing fatal outcomes and ensuring a complete resolution of symptoms.
